# Incidentaloma Discoveries in the Course of Neuroimaging Research

**DOI:** 10.1017/cjn.2018.397

**Published:** 2019-05

**Authors:** Emmanuel Stip, Jean-Philippe Miron, Marie Nolin, Geneviève Letourneau, Odette Bernazzani, Laurie Chamelian, Bernard Boileau, Mona Gupta, David Luck, Ovidiu Lungu

**Affiliations:** From the Department of Psychiatry, Université de Montréal, Montreal, Quebec, Canada; Institut universitaire en santé mentale de Montréal, Montreal, Quebec, Canada; Department of Psychiatry, Hôpital Maisonneuve-Rosemont, Montreal, Quebec, Canada; Department of Psychiatry, Centre hospitalier universitaire Sainte-Justine, Montreal, Quebec, Canada; Institut universitaire de gériatrie de Montréal, Montreal, Quebec, Canada

**Keywords:** Ethics, fMRI, Legal issues, Neuroethics, Neuroimaging, Neurological practice, Neurology - Education, Neuropsychiatry, Neurovascular, Psychiatry

## Abstract

Among healthy volunteers in psychiatric brain functional magnetic resonance imaging (fMRI) research studies, the prevalence of incidentalomas can be as high as 34%, of which 10% show clinical significance. An incidentaloma is a lesion found by coincidence without clinical symptoms or suspicion. Like lesions and other types of accidental findings, it is found in healthy individuals recruited to take part in psychiatric studies. The prevalence of these accidental findings among specific psychiatric populations remains unknown. However, a precise understanding of cerebral neuroanatomy, neuroradiological expertise, and an appropriate choice of fMRI exploration sequences will increase the sensitivity of identifying these accidental findings and enable researchers to address their clinical relevance and nature. We present recommendations on how to appropriately inform patients or participants of the accidental findings. Additionally, we propose specific suggestions pertaining to the clinical research setting aimed for investigators and psychiatrists. Unlike current articles pertaining to incidentaloma, the current report provides a distinct focus on psychiatric issues and specific recommendations for studies involving psychiatric patients.

## RÉSUMÉ

**Découvrir la présence d’incidentalomes dans le cadre de recherches en neuro-imagerie**. Chez les patients en santé participant de façon volontaire à des travaux de recherche du domaine de la psychiatrie faisant appel aux techniques d’IRM fonctionnelle, la prévalence d’incidentalomes peut atteindre jusqu’à 34 %. À noter par ailleurs que 10 % de ces incidentalomes se révèlent significatifs sur le plan clinique. Un incidentalome est une lésion détectée fortuitement qui ne présente pas de symptômes cliniques et qui ne suscite aucune suspicion. À l’instar d’autres lésions et d’autres observations de nature inopinée, on retrouve l’incidentalome chez des individus en santé ayant été recrutés pour participer à des études dans le champ de la psychiatrie. La prévalence de telles observations au sein de clientèles psychiatriques spécifiques demeure inconnue. Toutefois, une compréhension fine de la neuro-anatomie cérébrale et de l’expertise neuroradiologique, de même qu’un choix approprié de séquences d’IRM fonctionnelle, pourront faire en sorte d’augmenter la sensibilité de détection de ces lésions et ainsi permettre aux chercheurs d’évaluer leur nature et leur pertinence sur le plan clinique. Nous voulons présenter ici des recommandations permettant aux praticiens de révéler de façon appropriée aux patients ou aux participants la présence d’incidentalomes. Nous entendons aussi, pour le bénéfice des chercheurs et des psychiatres, faire des suggestions relatives au domaine de la recherche clinique. Contrairement aux articles récents explorant les incidentalomes, le présent compte-rendu mettra un accent particulier sur des enjeux psychiatriques et sur des recommandations spécifiques en vue d’études incluant une clientèle psychiatrique.

The frequent use of neuroimaging in psychiatry increases the chance that researchers will find incidental, asymptomatic lesions with potential clinical implications. However, the challenge is that most researchers conducting functional magnetic resonance imaging (fMRI) have insufficient neuroradiological training that would enable them to make such diagnoses. Consequently, identifying, interpreting, and managing these accidental findings in neuroimaging studies remains a controversial issue.^1^^,^^2^

In the current report, we briefly provide an overview of the nature and incidence of incidentalomas as reported in the research literature. We also discuss the possible health consequences of their discovery, particularly in the context of current procedures in neuroimaging research at medical, ethical, and legal levels.

Finally, we generate some recommendations aimed at establishing a pan-Canadian set of safety procedures that can be implemented at various research sites using magnetic resonance imaging (MRI or MR scanning).

## Method

### Inclusion Criteria

We conducted an electronic literature search using the keywords “incidental findings” OR “accidental discovery” OR “incidentaloma” OR “accidentaloma” using Google Scholar and PubMed in articles published from 1997 to 2016, without any restriction about language and age. From this search we identified a set of articles and commentaries (*n* = 24) addressing medical and ethical problems resulting from these discoveries (a summary of the articles not directly referenced can be found in Table 1).

**Table 1:** Summary of the articles included in the literature review

Weiner C.^1^	The authors argue that the possibility of incidental findings should be discussed with the participants and that their preferences regarding their disclosure and management should be assessed. Furthermore, they believe that findings with serious repercussions for which efficient treatments exist should be disclosed. Lastly, they remind us that research is costly and that putting too much funds in incidental findings management in a given study might affect its quality.
Berlin L.^19^	Commentary on patients’ autonomy regarding disclosure of incidental findings.
Mitka M.^20^	The author recommends “that those asked to conduct sequencing for a clinical indication also routinely evaluate and report other conditions, genes, and variants to the ordering clinician without seeking a patient’s or family’s preferences and without limits based on a patient’s age.” At odds with the Presidential Commission report.
Powell DK.^21^	The authors argue that radiological associations should publish pamphlets to patients and family doctors in order to educate about incidental findings.
Ells C, Thombs BD.^2^	The authors call for evidence-based guidelines on how to disclose incidental findings and argue that they should be anticipated and planned for in clinical and research settings.
Sexton SM.^13^	The author stresses on the importance of communication between physician and patient and shared decision making on which information should be disclosed and how.
Mccormick JB et al.^14^	New ways have to be developed to help patients make decisions that respect their needs and autonomy.
Kole J, Flester A.^11^	Patients should be provided with information about incidental findings in order to give an informed consent. That information should be provided by a radiologist, since they are the most knowledgeable on the subject.
Cramer SC et al.^17^	The authors devised a system allowing researchers using MRI technology to have access to a radiologist opinion when they have doubts regarding its applicability in routine research because of its cost.
Shoemaker JM et al.^18^	Researchers evaluated a systematic approach to incidental findings within a research network and found that providing neuroradiology interpretation of MRI scans and helping with clinical follow-up when indicated was cost-effective and suggest it could be adopted by other centers.
Lumbreras B et al.^22^	A meta-analysis describing the frequency of incidental findings across various imaging technologies and diagnosis.
Orme NM et al.^16^	Evaluation of research imaging by radiologists may lead to medical benefit in a small number of patients after identification and clinical action to address incidental findings.
Clayton EW.^23^	The potential utility of an incidental finding should be high in order to disclose it to a patient.
Wolf SM et al.^24^	Laws and institutions do not offer sufficient guidance to address the incidental findings problem.
Bos D et al.^9^	This study provides a rough estimate of abnormalities that usually are incidental findings on brain MRIs; very few of these abnormalities require intervention.

In order to illustrate the nature and the variability of these accidental findings, we included examples from our own studies – that is, focused on neural substrate of cognitive and motor processes or on issues related to schizophrenia. These studies were not designed to specifically identify anatomical lesions. Having consulted with a neuroradiologist while writing this manuscript, it is critical that the role of a neuroradiologist be included in protocol and the recommendations.

Information on follow-up and outcomes was not accessible or made available to us. The information on incidentaloma cases was handed over to the relevant physicians. In relation to follow-ups, every attending physician was responsible for their relevant cases once information was disclosed. As a result, outcomes were not included in our study. This study appreciates the difficulty of tracing back to identify case outcomes and, as a result, focuses on highlighting the lack of managing incidentaloma after a researcher discovers this.

### Prevalence

The prevalence of incidentaloma was found to vary between 1.7% and 6.0% for the most serious intracranial lesions, and this excluded sinus lesions, images of cerebral ischemia, and white matter hypersignals (the origin and meaning of which are by no means unequivocal).^3^^–^^8^ In the latest study from the Rotterdam group,^9^ the prevalence of at least one incidental finding of potential clinical relevance was 10% – meningiomas (2.5%), cerebral aneurysms (2.3%), arachnoid cysts (1.6%), and pituitary abnormalities (1.2%) being the most common.

It should be noted that the prevalence of these lesions varies across studies depending on the MRI sequences employed in each study. Consequently, these statistics may be underestimated in the general population^1^ compared with a representative sample of the general population.^10^ This could be due to the absence of contrast agents in research studies, the limited resolution of the anatomical MRI, and the absence of angio-MRI sequences in research protocols – which is of particular relevance in detecting aneurysms, arteriovenous malformations, and certain tumors.

### Consequences of Incidental Findings

There are three types of consequences resulting from the discovery of incidentalomas in research studies: clinical, ethical, and legal. Most such discoveries are made retrospectively – a few months or years after the research participant has undergone radiological investigation. From a clinical point of view, early detection of brain tumors is important in assuring a successful surgical or chemotherapeutical intervention; therefore, the delay in detecting incidentalomas in research studies could potentially compromise participants’ health outcomes. This issue highlights the urgency of implementing systematic monitoring of these lesions.^1^^,^^2^

To date, the ethics surrounding incidentalomas are complex. The US Presidential Commission report provides a lengthy overview of the ethical issues.^1^ On the one hand, it is clear that if a serious tumor is detected or if its evolution has the potential to become a serious medical threat, such as an aneurysm, the support for the patient should not be delayed. On the other hand, guidelines are ambiguous in relation to cases involving a small lesion with no potential risk of harm. This raises the question if and how professionals should inform participants. Informing research volunteers that they have a cerebral lesion may cause unnecessary anxiety and lead to unwanted social consequences (insurance, loans, job loss due the potential epileptogenic risk of the lesion). Furthermore, it is necessary to distinguish between healthy volunteers and psychiatric patients since the ethical responsibilities differ by type of research participant.

Finally, there are potential legal consequences of incidentalomas particularly concerning the procedures in place to manage them. If a patient had a lesion resulting in serious health repercussions or even death, which was not announced, she or her family might legitimately seek damages on the grounds that the outcome was a foreseeable risk of research participation. The absence of procedures in place to report or diagnose brain lesions may trigger the forced implementation of risk management strategies.

### Illustration

**Figure 1:**
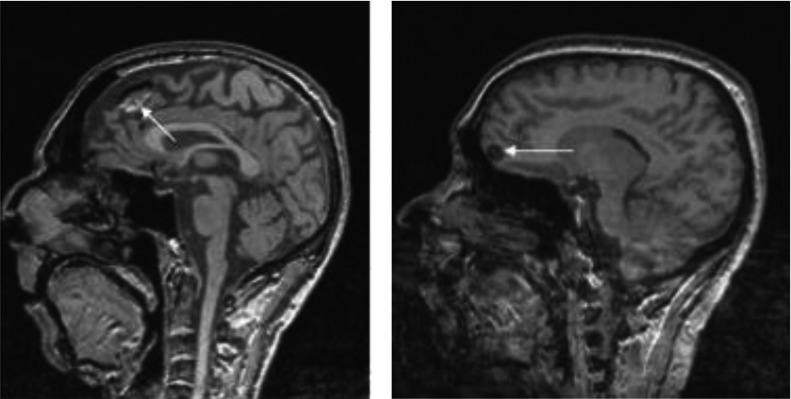
Sagittal T1 sections: Heterogeneous frontal mass (in hyper- and hyposignals) in the image on the left and a homogeneous hyposignal nodular lesion on the right. These images could correspond to tumor lesions – further investigation is needed.

**Figure 2:**
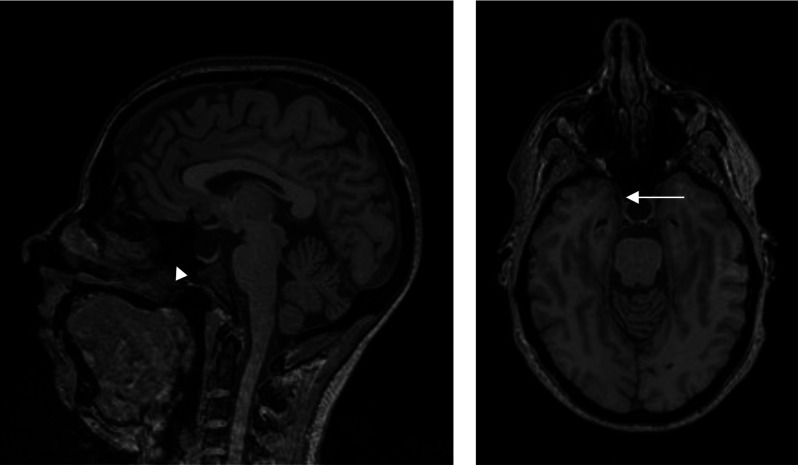
Sagittal and axial T1 sections: Increased volume of the sella turcica (see arrow) occupied by fluid with pituitary tissue (arrow head) pressed against the sella turcica: cystic pituitary lesion or empty sella syndrome.

**Figure 3:**
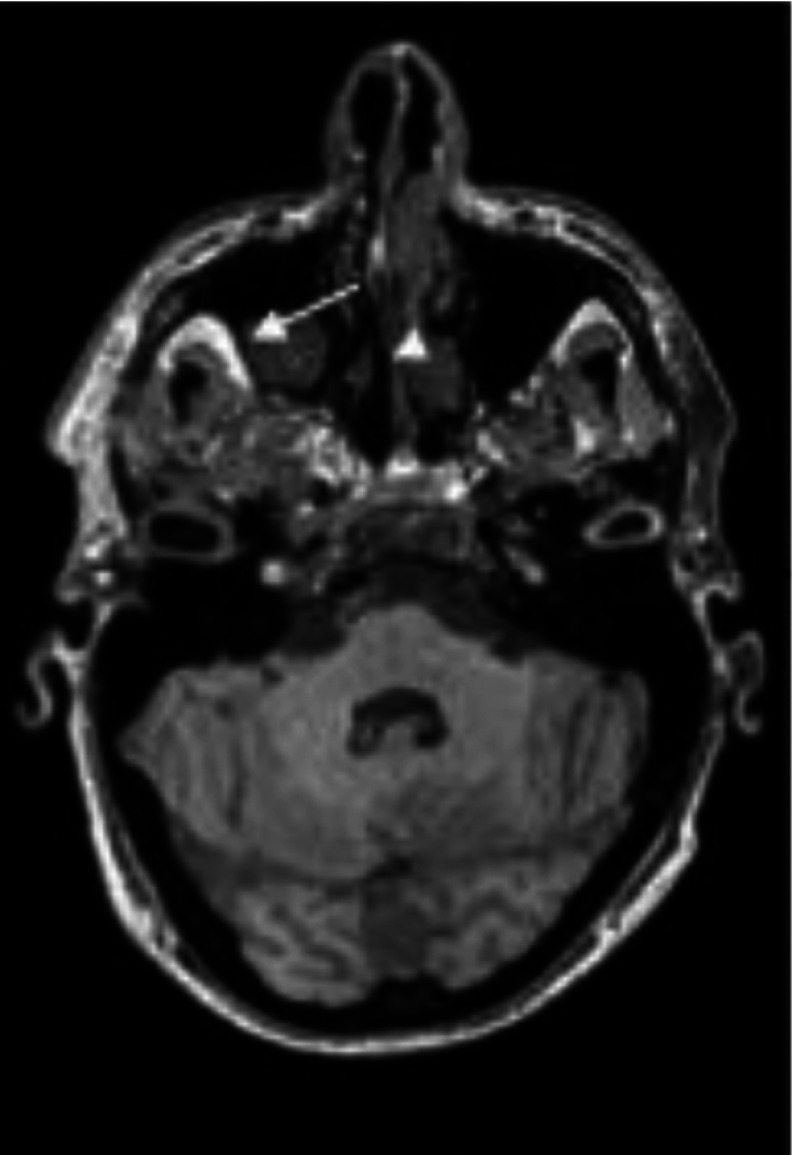
Polyploid tissue formation in the right maxillary sinus (arrow) with hypertrophy and of the lining of the left nasal cavity (arrowhead).

To simply give the reader a visual representation of the problem, we present the images in Figures 1–3 where accidentalomas were highlighted during some research projects conducted by our group. The research projects concerned healthy subjects and patients with schizophrenia who were investigated for relationships between cerebral activation and cognitive or emotional functioning.

## Discussion

During clinical studies, images are often only analyzed using computer software, and it is rare for them to be seen by a neuroradiologist. Given the growing number of research protocols, the prevalence of incidentalomas, and the fact that such images are usually seen by researchers who are not experts in the field of neuroradiology,^11^ raises questions about what level of sensitivity in detection procedures ought to be required in order to properly protect the welfare of research participants.

Including details about access to neuroradiological expertise in the research protocols using MRI may lead to a greater number of participants benefiting from curative (in the case of tumors) or preventive interventions (aneurysms >7 mm present an elevated subarachnoid hemorrhage risk).^12^ Currently, institutions such as the National Institutes of Health and the institutional review boards require that all MRI scans be seen by a neuroradiologist.^12^ However, procedures vary depending on the research site, whereby the specialist may not be required to present a report to accompany the images or the images are first analyzed by the scientists themselves (often with no medical or radiological training), who then send the images to a radiologist if anything appears suspicious. Furthermore, among the sites where all images are reviewed, there are delays of several weeks or months between the time the MRI is performed and the time the images are analyzed.

It is important to note that the choice of MRI sequences used in research is limited. In that, the most commonly used anatomical sequence in fMRI studies uses the T1-weighted contrast, which lacks the sensitivity and specificity of revealing lesions.

## Recommendations

As proposed by Hoggard,^7^ T2-weighted axial sections of 3–4 mm thickness or FLAIR inversion recovery sequences could clearly increase the level of detection. Therefore, we could adapt the MRI sequences to maximize the discovery of lesions that may go undetected with T1 weighting.

Although study participants presently give their consent after receiving information about the protocol and the possible risks inherent to MRI, they should also be informed of the possibility and implications of finding a brain anomaly and the opportunity to consider this matter carefully before agreeing to volunteer.^2^^,^^13^^,^^14^ They should be informed and made aware that the purpose of the study is not to identify the asymptomatic brain lesions.

We concur with Nelson^15^ in that research funding organizations should require that (1) all images are reviewed by a neuroradiology council, (2) researchers use some of their budget for this council, and (3) the images are archived for at least a few years. An alternative could be to treat such radiological assessments as quasi-health services, to be funded publicly on the grounds that the researchers collecting the images are performing *volens nolens* (non-clinical) screening service for the general public.

To improve the power of detection, research managers should ensure their students obtain adequate training in radiological neuroanatomy and develop a method of exploring the gross anatomical images before submitting them to software and statistical processing. As part of a research protocol, basic education (e.g., 50 h) should be provided by a professor in neuroradiology in order for students to be better able to distinguish and determine the results of a CT scan, an MRI scan, an angiogram, and X-rays of the brain, spinal column, face, neck, and peripheral nerves. Additionally, research managers and students should also have adequate training in social skills in order to better collaborate with neuroradiologists and colleagues with expertise in other specialties with different levels of education and responsibility, such as nurses and medical support staff. How to fund such additional obligations is an important question that has received relatively little attention. The analysis will vary depending on the type of health care system one has. Some scientists worry that putting too much effort in diagnosing and managing could be costly and reduce the research quality of a given study.^1^^,^^16^^,^^17^ In the absence of a unified recommendation as a part of the condition to conduct imaging research, the subjects should be informed how their images will be analyzed, and that should their results warrant further investigation, an appropriate specialist will be consulted.

## Conclusion

One possible benefit of participation in clinical research is having more assiduous medical care. This should be made clear alongside the relative risks of false positives that are part of neuroimaging research. The consequences of finding potentially dangerous, time-consuming, worrying lesions are not negligible. Our recommendation that radiological review and potential neurology follow-up care must be put in this context. Our recommendations attempt to address the problem of incidental findings in the context of current practice of neuroscience research, be it done with healthy volunteers or patient populations. We believe that professional and medical associations, such as the Canadian Psychiatry Association, the Canadian Neurological Sciences Federation, and the Canadian College of Neuropsychopharmacology, should consider these recommendations and implement them as a measure of best practice among clinicians and researchers who use brain imaging.

## In Practice

In case of an incidental finding, we propose the following approach:

1. Researcher(s) to inform the Principle Investigator (PhD or MD) and researcher(s) to forward anonymized images to a radiologist for review.
2. Radiologist (part of, or hired by, the team) to review anonymized images and generate a non-clinical descriptive summary, advising whether the findings seen warrant follow-up by a physician in a clinical setting and/or psychiatrist if it is a patient’s image.
3. Principal Investigator to inform the participant that the findings of their image require investigation by a neurologist.
4. Participants are given the option of making their own arrangements for follow-up with a physician at an external clinic.
5. If participants are not able to make arrangements at an outpatient clinic, the services of a neurologist affiliated with the research lab should be offered for the follow-up. With the participant’s consent, the radiologist’s summary will be made available to the neurologist. Appropriate and detailed consent is essential regardless of the actual approach undertaken, in order to ensure subjects make an informed decision.
6. With the participant’s consent, a copy of the anonymized images and the descriptive summary, along with a letter of introduction, can be forwarded to a physician who is following the participant at an outpatient clinic and/or to the psychiatrist.

For example, the Unité de Neuroimagerie Fonctionelle, a research facility in Montreal (see Table 2; www.unf-montreal.ca), suggests the following script to be used as a model for researchers when there has been a coincidental finding:

“Recently, you participated in a research project involving a magnetic resonance scan. An unusual feature was noticed on some of the images, so we asked a radiologist (a medical doctor who is qualified to read these images) to review the images. The radiologist has recommended that you follow up with a proper medical examination, since the research images may not include all of the information needed to make a clinical diagnosis. If you wish, a copy of the images can be forwarded to your family doctor or any other physician you would like to designate. If you do not have a family doctor, we can make arrangements for you to be seen by a neurologist associated with our Research Unit.”

**Table 2:** Illustrative script for researchers when there has been a coincidental finding

Possible questions	Possible answers
What should I say to my doctor?	You participated in a research project and some MR images were acquired. On reviewing the images, something unusual was noted. A radiologist reviewed the images and recommended a follow-up with your physician
Can you tell me what is wrong? What did you see? I want more details. Is it a tumor, blood vessel, multiple sclerosis…? How big is it? Where is it? Can you please contact my doctor?	A copy of the images can be forwarded to your doctor. Once your doctor has had a chance to see you and review the images, he or she will be able to give you more information.
Do I really need to go and see a doctor? Do I need to see a specialist or a regular doctor? What should I do now? Where should I go? (clinic, hospital, emergency room) When do I need to see a doctor? (today vs. next week) Refer me to the doctor (radiologist) who has already looked at my images. I cannot get an appointment within 6 months. Can I be followed here? Why can’t I see a doctor here? This hospital must be full of doctors and nurses.	A radiologist has recommended that you be followed. Perhaps it would be best to follow that recommendation. Ultimately the choice is yours. The radiologist who reviewed your films is not available to follow your case; you need to see a family doctor/specialist who can review your case and follow you up, if required.
